# Immunological and virologic outcomes of people living with HIV in Guangxi, China: 2012-2017

**DOI:** 10.1371/journal.pone.0213205

**Published:** 2019-03-01

**Authors:** Xueying Yang, Xiaoming Li, Shan Qiao, Quan Zhang, Zhiyong Shen, Yuejiao Zhou

**Affiliations:** 1 Department of Health Promotion, Education, and Behavior, South Carolina SmartState Center for Healthcare Quality (CHQ), Arnold School of Public Health, University of South Carolina, Columbia, SC, United States of America; 2 Guangxi Center for Disease Control and Prevention, Nanning, Guangxi, People’s Republic of China; Centers for Disease Control and Prevention, UNITED STATES

## Abstract

**Background:**

Limited data are available on clinical outcomes of people living with HIV (PLWH) in China, especially after the implementation of the 2016 national treatment guideline. The objective of the current study is to examine the treatment patterns, clinical outcomes and their associated factors among PLWH in Guangxi, China before and after this new guideline.

**Methods:**

Data from three community-based projects conducted at different time points over a period of six years (2012–2017) in Guangxi were analyzed in our study. The interviewer-administered questionnaire was used for data collection. Measures of clinical outcomes were retrieved from the patients’ medical records. Descriptive analysis was employed to display treatment patterns and the time trends of clinical outcomes. Chi-square test or ANOVA was used to compare the differences in background characteristics and treatment history between different levels of clinical outcomes.

**Results:**

Among the pooled sample of 4224 participants, 77.3% were receiving antiretroviral therapy (ART), the median CD4 count was 328 cells/mm^3^, and 82.5% were virally suppressed. An increasing trend in both ART coverage (from 72.1% to 91.2%) and CD4 count (from 318 cells/mm^3^ to 357 cells/mm^3^) was observed over time in the three samples, while rates of viral suppression did not show a similar trend. A number of socio-demographic characteristics (e.g., female gender, younger age, Han ethnicity, and employment) and treatment-related variables (e.g., longer durations of HIV diagnosis and ART uptake, lower prevalence of comorbidity, fewer treatment interruptions, and more knowledge on ART) were associated with improved clinical outcomes.

**Conclusions:**

We observed a high rate of viral suppression and increasing trends in ART coverage and CD4 count over six years in Guangxi, China. However, suboptimal clinical outcomes continue to be a problem, particularly among some subgroups of PLWH. Future clinical management strategies should be tailored for PLWH with different sociodemographic characteristics and treatment trajectories.

## Introduction

Antiretroviral therapy (ART) has been used worldwide and achieved great success in HIV treatment and prevention since its advent in the 1990s. The preventive benefits of the early use of ART in asymptomatic HIV infection have been proved with clinical evidence in the last decade [1, 2]. Furthermore, increasing evidence also documented various benefits of the early use of ART in substantially reducing AIDS-related morbidity and mortality [1, 3–6].

Based on the clinical evidence, World Health Organization (WHO) updated its guideline and recommended ART to all PLWH regardless of their CD4 counts [7]. The Joint United Nations Programme on HIV/AIDS (UNAIDS) launched its ambitious 90-90-90 targets in 2014: 90% of the PLWH knowing their HIV status, 90% of the diagnosed PLWH receiving ART, and 90% of the PLWH on ART achieving viral suppression [8]. However, across the globe, among the estimated 36.9 million PLWH, only 59% of them were receiving ART and 47% achieved viral suppression among those on ART in 2017 [9]. In terms of specific regions, the percentage of PLWH who were receiving ART ranged from 29% (Middle East and North Africa) to 76% (western and central Europe and North America); meanwhile, the percentage of viral suppression among those PLWH on ART ranged from 22% (Middle East and North Africa) to 65% (western and central Europe and North America) [10]. It’s noteworthy that most countries are still far behind to achieve the UNAIDS 90-90-90 target, including China.

The HIV epidemic in China is still alarming. It is estimated that the accumulative number of PLWH has reached 1.25 million in 2018, and the reported number of PLWH is 850,000 [11]. Following WHO guidelines, China revised their ART guideline in 2014 and broadened the ART eligibility criteria by increasing CD4 threshold for the provision of free ART from ≤350 cells/mm^3^ to ≤500 cells/mm^3^ [12]. In 2016, the national ART guideline was revised again, whereby all PLWH were recommended for ART regardless of CD4 count [13].

Both CD4 count and HIV viral load are critical treatment indicators for determining the clinical stages of HIV infection, for evaluating the efficacy of treatment, and for changing the medication when necessary [14, 15]. However, according to a 2016 meta-analysis, only around 40% of PLWH were on ART and 36% of those on ART achieved viral suppression in China [16]. The immunological outcome was also not satisfactory, with the median CD4 count ranging from only 118 to 182 cells/mm^3^ [17–19]. The relatively better virologic outcomes were reported in some studies, with the rates of viral suppression ranging from 79.2% to 87.9% [18–21]. However, most of these studies defined viral suppression as HIV RNA <1000 copies/ml, which is much higher than the cutoff used in recent literature (HIV RNA <50 copies/ml). Furthermore, all these studies were conducted before 2016 and might not reflect the situation after the implementation of the latest national treatment guideline. In addition, most of the existing studies were conducted based on data from the nationwide National Free Antiretroviral Treatment Program [17], certain urban sentinel surveillances [18, 21], or among specific key populations (e.g., injecting drug users) [19, 20]. The participants in those studies were mostly receiving ART in the urban areas, which may not be representative of situations in other areas in China with a large rural population, such as Guangxi Zhuang Autonomous Region (Guangxi) of China.

As one of the regions with the fastest growing HIV epidemic in China, Guangxi has reported a total of 124,282 HIV/AIDS cases by the end of 2017, indicating a 78.7% increase since June 2011 (69,548 HIV/AIDS cases) and placing Guangxi second among 31 Chinese provinces in terms of HIV seropositive cases [22, 23]. The HIV epidemic in Guangxi is largely characterized as a rural phenomenon with many PLWH being rural residents, having low socio-economic status, and being infected mainly through sexual contacts [24]. Guangxi has scaled up ART since 2005 and the number of individuals receiving free ART has increased dramatically since June 2010 under the Guangxi government’s anti-HIV programme [25]. But till now, only a few studies reported the immunological and virologic outcomes of PLWH in Guangxi [26].

The primary objective of our study, therefore, is to examine the treatment patterns and clinical outcomes before and after the implementation of the latest national treatment guideline using data from three samples in Guangxi. In addition, we compared the differences in background characteristics and treatment history between different levels of immunologic and virologic outcomes.

## Materials and methods

### Study site and participants

Data used in the current study were derived from three different community-based projects conducted in Guangxi, China. All the participants in the three projects were recruited from community-based treatment settings, following similar inclusion and exclusion criteria [27, 28].

### Study one

The first study was a cross-sectional study which aimed to understand the health status (e.g., quality of life, mental health) of PLWH in Guangxi [27]. The data in this study were collected from October 2012 to August 2013. In this project, two cities and ten rural counties that had the largest cumulative number of reported HIV/AIDS cases were selected as the study sites. The main inclusion criteria were aged ≥18 years old and with a confirmed HIV diagnosis. With an appropriately 10% refusal rate, 3,002 PLWH were recruited. A total of 2,987 participants were included in the data analysis after removing 15 uncompleted questionnaires.

### Study two

The second study was a theory-based HIV disclosure intervention trial and primarily aimed to assist parents living with HIV/AIDS to plan or make a developmentally appropriate disclosure of their HIV status to their seronegative children (aged 6–15 years). The baseline data of the intervention project were collected from July 2013 to March 2014. In this project, eight cities and eight rural counties with the largest cumulative number of reported HIV/AIDS cases in 2012 were selected as study sites. We further identified all the HIV clinics with at least 200 HIV/AIDS cases in these urban districts and rural townships, and randomly selected 40 of them to participate in the intervention trial. We then randomly invited about 20 eligible HIV-infected parents from each clinic to participate in the trial. The main inclusion criteria for parents include: 1) at least 18 years of age; 2) a confirmed diagnosis of HIV or AIDS; 3) living with at least one child 6 to 15 years of age; and 4) having not disclosed their HIV status to their children. A total of 791 participants completed the baseline survey, with the refusal rate being around 5%.

### Study three

The third study, where the data were collected after the implementation of the latest national ART guideline (i.e., between September 2016 and November 2017), was an exploratory longitudinal study and aimed to investigate the mechanism of the effects of HIV disclosure on clinical outcomes. In this project, we selected two cities (urban centers) and eight rural counties with the largest number of reported HIV/AIDS cases by 2014 as our study sites. With a similar approach in the first and second studies, we identified HIV clinics in the urban districts and rural townships which has at least 200 HIV/AIDS cases. We randomly selected 40 these clinics (8 urban clinics and 32 rural clinics) to participate in our study. The local research team randomly selected about 10 PLWH who were diagnosed since January 2014 (“recently diagnosed”) from each clinic. The main inclusion criteria of this project were similar to the first two projects, with the exception that only those “recently diagnosed” PLWH were included. A total of 446 participants completed the baseline survey.

### Data collection

The survey procedures of the three projects were similar and had been described elsewhere [27]. Briefly, data were collected through an interviewer-administered survey in all three samples. The participants’ ART status, CD4 count, and viral load were retrieved from their medical records. All participants provided written informed consent before participating in the study. The first study protocol was approved by the Institutional Review Boards at Wayne State University in the United States and Guangxi Center for Disease Control and Prevention (CDC) in China. The second and third study protocols were approved by the Institutional Review Boards at University of South Carolina in the United States and Guangxi CDC in China.

### Measures

#### Socio-demographics

Participants were asked about their socio-demographics, which include gender, age, education level (i.e., years of formal schooling), employment status (e.g., full-time job, part-time job, no job), marital status (e.g., single, married, living with boyfriend or girlfriend, divorced, or widowed), monthly household income in Chinese Yuan (CNY) (e.g., 1000–1999, 2000–2999, 3000–3999), and HIV transmission mode (e.g., sexual intercourse with spouse/regular partners/casual partners, commercial sex, blood transfusion, injection drug use).

#### Immunologic and virologic outcomes

The most recent CD4 count and the most recent plasma viral load were retrieved from patients’ medical records. For the purpose of data analysis in the current study, CD4 count was categorized into three groups (≤350 cells/mm^3^, 350–500 cells/mm^3^ and >500 cells/mm^3^), using the cutoffs in different versions of the national treatment guidelines [12, 13, 29]. Viral suppression was defined as HIV RNA <50 copies/ml.

#### Other HIV and treatment-related measures

Duration of HIV diagnosis and ART uptake were calculated based on the patients’ self-report. The co-morbidity of HIV infection was also based on self-report and defined as co-infection with specific bacteria or viral infections, including Hepatitis A, Hepatitis B, Hepatitis C, Tuberculosis, Syphilis, Gonorrhea, and genital Herpes Simplex Virus-2. Treatment interruption was defined as not taking ART medication in the past month. The ART-related knowledge was assessed with a 12-item scale. The scale was developed based on existing literature [30–32] and qualitative interview by the research group, comprised of psychologists and local health care providers. Each item was scored on a 4-point Likert scale ranging from “1 = strongly disagree” to “4 = strongly agree”. After appropriate reverse-scoring of certain items, a composite score was calculated by summing up the responses to all the items in the scale.

### Statistical analysis

Descriptive analysis was employed to describe the socio-demographic characteristics, treatment history, the immunologic (CD4 counts) and virologic outcomes (viral load) in three different samples. Chi-square test or ANOVA was used to compare the differences in background characteristics and treatment history between different levels of immunologic (CD4 count: ≤350 cells/mm^3^, 350–500 cells/mm^3^, and >500 cells/mm^3^) and virologic outcomes (HIV RNA<50 copies/ml vs ≥50copies/ml). SAS 9.4 was used for all the statistical analyses.

## Results

### Sample characteristics

As presented in Table 1, a total of 4224 PLWH participated in three studies (n = 2987 in study one, n = 791 in study two, and n = 446 in study three). Among the pooled sample, around two-third (62.3%) were male; the mean age was 41.6 years (SD = 11.1); the majority of them (69.5%) were of Han ethnicity; the average years of schooling was 6.9 years (SD = 2.8); 42.8% had a full-time job; 71.7% were currently married or living with a boyfriend or girlfriend; only 19.6% had a monthly household income of more than 2000 CNY (appropriately equal to US$ 291). Among the participants who identified the route of infection, the majority (82.1%) reported that they acquired HIV through sexual intercourse.

**Table 1:** Background characteristics of three samples of PLWH in Guangxi.

	Pooled sample (2012–2017)	Sample 1 (2012–2013)	Sample 2 (2013–2014)	Sample 3 (2016–2017)
**N**^**a**^	**4224**	**2987**	**791**	**446**
***Socio-demographic***				
Gender				
Male	2598 (62.3)	1876 (62.8)	420 (57.0)	302 (68.0)
Female	1570 (37.7)	1111 (37.2)	317 (43.0)	142 (32.0)
Age (mean±SD, years)	41.6 ±11.1	42.5 ±12.8	38.0 ±5.6	41.6 ±8.5
Ethnicity				
Han	2889 (69.5)	2109 (71.7)	513 (69.9)	267 (60.7)
Others	1267 (30.5)	873 (29.3)	221 (30.1)	173 (39.3)
Education (mean±SD, years)	6.9±2.8	7.0 ±3.0	6.9±2.5	6.2±2.4
Job				
No job	1058 (25.2)	800 (26.9)	155 (19.8)	103 (23.3)
Part-time	1345 (32.0)	992 (33.4)	262 (33.4)	91 (20.5)
Full-time	1798 (42.8)	1182 (39.7)	367 (46.8)	249 (56.2)
Marital status				
Single/Separated	452 (10.9)	386 (13.2)	15 (1.9)	51 (11.5)
Married/Living with boyfriend or girlfriend	2975 (71.7)	2011 (68.9)	613 (77.6)	351 (79.4)
Divorced/Widowed	722 (17.4)	520 (17.8)	162 (20.5)	40 (9.1)
Household's monthly income (CNY)				
< 2000	3369 (80.4)	2442(82.6)	684 (86.5)	243 (54.9)
≥2000	823 (19.6)	516(17.4)	107 (13.5)	200 (45.2)
HIV transmission mode				
Sexual intercourse	2880 (82.1)	1962(79.7)	543 (82.3)	375 (94.2)
Transfusion/needling sharing	630 (17.9)	501 (20.3)	117 (17.7)	12 (3.1)
***HIV-related characteristics***				
Duration of HIV diagnosis (mean±SD, months)	42.1±27.6	43.6±29.3	48.6 ±30.6	19.2 ±10.3
The most recent CD4 count				
Median (IQR^b^)	328 (272)	318 (263)	347 (259)	357 (328)
≤350	2198 (54.2)	1599(56.0)	385 (50.5)	214 (49.3)
350–500	994 (24.5)	697(24.4)	198 (26.0)	99 (22.8)
>500	861 (21.2)	560(19.6)	180 (23.6)	121 (27.9)
HIV RNA <50 copies/ml	1836 (82.5)	1341(81.6)	356 (85.8)	139 (82.7)
Any co-morbidity (Yes)	699 (16.6)	470 (15.7)	168 (21.2)	61 (13.7)
***Treatment history***				
Whether on ART				
Yes	3251 (77.3)	2146 (72.1)	703 (89.0)^c^	402 (91.2)
No	956 (22.7)	830 (27.9)	87 (11.0)	39 (8.8)
Duration of ART (mean±SD, months)	34.8±23.0	36.7±23.8	36.9±26.4	18.9±11.3
Treatment interruption (Yes)	96 (3.5)	75 (3.6)	21 (3.1)	NA
Knowledge on ART (mean±SD)	38.3±3.8	38.2±3.7	NA	39.1±4.7

^a^ The number of participants for some of the variables did not add up to the total sample size because of missing data

^b^ IQR: Interquartile Range

^c^ Self-reported ART status.

With regard to HIV-related characteristics, the average duration since their HIV diagnosis was 42.1 months (SD = 27.6); around one sixth (16.6%) of the participants had at least one kind of co-morbidity (e.g., Hepatitis or Syphilis). The three samples were similar in terms of background characteristics (Table 1), except that in sample 3, a larger proportion (45.2%) of the participants had a high monthly household income (≥2000 CNY or US$ 291), and the duration of HIV diagnosis was much shorter than PLWH in the first two samples, which reflected the inclusion of only recently diagnosed parents in the third sample.

### Treatment patterns

As shown in Table 1, 77.3% of the pooled sample were receiving ART, and among those who were on ART, the average duration of ART initiation was 34.8 months (SD = 23.0). The proportion of PLWH on ART increased from 72.1% in 2012–2013 (sample 1) to 89.0% in 2013–2014 (sample 2) and 91.2% in 2016–2017 (sample 3). The duration of ART varied in the three samples, with the participants in sample 3 having a much shorter ART treatment history (18.9 months) than the other two samples because of the inclusion criteria in study 3. A small proportion (3.5%) of participants had experienced treatment interruptions based on pooled available data from sample 1 and 2. The average score of the ART-related knowledge scale was 38.3 (SD = 3.8) based on the available data from sample 1 and 3.

### Immunological and virologic outcomes

The data on CD4 count were available from 95.95% of the pooled sample (95.61% in sample 1, 96.46% in sample 2, and 97.31% in sample 3) with a median value of 328 cells/mm^3^. The CD4 count increased from 318 cells/mm^3^ in 2012–2013 (sample 1) to 347 cells/mm^3^ in 2013–2014 (sample 2) and 357 cells/mm^3^ in 2016–2017 (sample 3). In the pooled sample, over half of them (54.2%) were having CD4 counts ≤350 cells/mm^3^, one fourth of them (24.5%) having CD4 counts between 350–500 cells/mm^3^, while around 20% of them having CD4 counts >500 cells/mm^3^. The proportion of PLWH with CD4 counts ≤350 cells/mm^3^ decreased from 56.0% in 2012–2013 to 50.5% in 2013–2014 and 49.3% in 2016–2017, while those with CD4 counts >500 cells/mm^3^ increased from 19.6% in 2012–2013 to 23.6% in 2013–2014 and 27.9% in 2016–2017. (Table 1)

Data on viral load were available from 52.7% of the pooled sample (55.01% in sample 1, 54.47% in sample 2, and 37.67% in sample 3). Among those participants, 82.5% had viral suppression. Similar rates were observed in the three individual samples, although the rates in sample 2 (85.8%) and sample 3 (82.7%) were both slightly higher than that in sample 1 (81.6%). (Table 1)

### Factors associated with clinical outcomes

In terms of immunological outcome, PLWH who were female (p < .0001) and younger (p<0.01) were more likely to have a higher CD4 count than their counterparts in all three samples. In sample 1, those PLWH who had no job or only part-time job had a lower CD4 count (p<0.05), while this relationship was not significant in the other two samples. In addition, PLWH who were diagnosed for a longer time (p<0.001), being on ART (p<0.001), being virally suppressed (p = 0.01), and lower prevalence of comorbidity (p = 0.003) were having higher CD4 counts in sample 1, while the latter two factors were not significant in the other two samples. The participants who had been on ART for a longer time were more likely to have a higher CD4 count (p<0.05) in all three samples. (Table 2)

**Table 2:** Associations between socio-demographic, treatment history and CD4 counts among three samples of PLWH in Guangxi.

	Sample 1 (N = 2856)				Sample 2 (N = 763)				Sample 3 (N = 434)			
**CD4 count (cells/mm**^**3**^**)**	≤350	350–500	>500	P	≤350	350–500	>500	P	≤350	350–500	>500	P
**N (%)**^**a**^	1599 (56.0)	697 (24.4)	560 (19.6)		385 (50.5)	198 (26.0)	180 (23.6)		214 (49.3)	99 (22.8)	121 (27.9)	
***Socio-demographic***												
Gender												
Male	1081 (67.6)	392 (56.2)	305 (54.5)	**< .0001**	256 (66.5)	96 (48.5)	82 (45.6)	**< .0001**	163 (77.6)	61 (62.2)	69 (57.0)	**0.0002**
Female	518 (32.4)	305 (43.8)	255 (45.5)		129 (33.5)	102 (51.5)	98 (54.4)		47 (22.4)	37 (37.8)	52 (43.0)	
Age (mean±SD, years)	43.9 ±12.9	41.4±12.7	39.1 ±11.5	**< .0001**	38.4 ±5.4	37.5±5.8	36.9 ±5.3	**0.01**	43.2 ±8.0	39.6 ±8.9	40.4±8.5	**0.0005**
Ethnicity												
Han	1111 (69.7)	490 (70.4)	392 (70.0)	0.93	248 (69.1)	130 (71.0)	118 (71.1)	0.85	118 (55.9)	62 (63.3)	79 (65.3)	0.19
Others	484 (30.3)	206 (29.6)	168 (30.0)		111 (30.9)	53 (29.0)	48 (28.9)		93 (44.1)	36 (36.7)	42 (34.7)	
Education (mean±SD, years)	6.9 ±3.0	7.1 ±3.0	7.1 ±2.9	0.13	6.8±2.5	6.8±2.6	7.2 ±2.6	0.17	6.1 ±2.2	6.2 ±2.5	6.5±2.6	0.30
Job												
No job	452 (28.4)	167 (24.1)	144 (25.7)	**0.04**	70 (18.4)	40 (20.4)	38 (21.2)	0.62	55 (25.8)	22 (22.2)	25 (20.7)	0.37
Part-time	541 (34.0)	227 (32.8)	175 (31.3)		136 (35.7)	58 (29.6)	60 (33.5)		39 (18.3)	18 (18.2)	32 (26.5)	
Full-time	599 (37.6)	299 (43.2)	241 (43.0)		175 (45.9)	98 (50.0)	81 (45.3)		119 (55.9)	59 (59.6)	64 (52.9)	
Marital status												
Single/Separated	211 (13.5)	86 (12.7)	68 (12.4)	0.18	10 (2.6)	2 (1.0)	3 (1.7)	0.60	29 (13.6)	12 (12.2)	10 (8.3)	0.45
Married/Living with boyfriend or girlfriend	1051 (67.2)	476 (70.0)	397 (72.5)		292 (75.8)	157 (79.3)	143 (79.9)		162 (76.1)	80 (81.6)	99 (81.8)	
Divorced/Widowed	301 (19.3)	118 (17.4)	83 (15.2)		83 (21.7)	39 (19.7)	33 (18.4)		22 (10.3)	6 (6.1)	12 (9.9)	
Household's monthly income (CNY)												
< 2000	1312 (83.0)	562 (81.3)	453 (81.2)	0.47	337 (87.5)	162 (81.8)	159 (88.3)	0.11	120 (56.1)	53 (54.1)	63 (52.1)	0.77
≥2000	268 (17.0)	129 (18.7)	105 (18.8)		48 (12.5)	36 (18.2)	21 (11.7)		94 (43.9)	45 (45.9)	58 (47.9)	
HIV transmission mode												
Sexual intercourse	1073 (81.8)	456 (79.2)	358 (77.0)	0.06	257 (78.8)	138 (85.2)	127 (86.4)	0.07	182 (96.3)	84 (96.7)	99 (97.1)	0.82
Transfusion/needling sharing	239 (18.2)	120 (20.8)	107 (23.0)		69 (21.2)	24 (14.8)	20 (13.6)		7 (3.7)	2 (2.3)	3 (2.9)	
***HIV-related characteristics***												
Duration of HIV diagnosis (mean±SD, months)	40.2±28.3	46.3±28.5	50.5±30.9	**< .0001**	43.2±29.9	52.4±30.4	55.5±29.6	**< .0001**	17.8±9.7	18.6±9.6	22.2±11.4	**0.0016**
HIV RNA <50 copies/ml	751 (79.6)	325 (82.3)	260 (87.3)	**0.01**	160 (82.0)	89 (87.3)	82 (89.1)	0.22	63 (77.8)	33 (89.2)	43 (86.0)	0.24
Any co-morbidity (Yes)	283 (17.7)	84 (12.1)	87 (15.5)	**0.003**	95 (24.7)	40 (20.2)	30 (16.7)	**0.08**	35 (16.4)	13 (13.1)	11 (9.1)	0.17
***Treatment history***												
Whether on ART												
Yes	1262 (79.2)	473 (68.1)	356 (63.8)	**< .0001**	349 (90.7)	179 (90.9)	153 (85.0)	0.09	195 (92.0)	87 (89.7)	111 (93.3)	0.63
No	331 (20.8)	222 (31.9)	202 (36.2)		36 (9.4)	18 (9.1)	27 (15.0)		17 (8.0)	10 (10.3)	8 (6.7)	
Duration of HIV treatment (mean±SD, months)	33.4±21.7	39.9±24.4	45.9±28.1	**< .0001**	32.8±25.0	37.8±25.3	46.2±29.2	**< .0001**	17.3±9.6	17.9±9.3	21.1±10.6	**0.01**
Treatment interruption (Yes)	53 (4.3)	11 (2.4)	9 (2.6)	0.10	15 (4.5)	4 (2.3)	2 (1.4)	0.14	NA	NA	NA	
Knowledge on HIV treatment (mean±SD)	38.2±3.6	38.2±3.9	38.3±3.5	0.79	NA	NA	NA		39.1±4.9	38.3±4.1	39.6±4.9	0.13

^a^ The number of participants for some of the variables did not add up to the total sample size because of missing data

With respect to virologic outcome, the study 3 participants who were of Han ethnicity had a higher rate of viral suppression (p = 0.01); the study 1 participants who did not have a full-time employment (e.g., had no job or part-time job) (p = 0.007) or had no stable partner (e.g., not married, not living with boyfriend or girlfriend) (p = 0.04) reported a lower level of viral suppression. A longer duration of HIV diagnosis was associated with viral suppression in both samples 1 and 3 (p<0.001). Being on ART (p<0.05) and receiving ART for a longer time (p<0.001) were positively associated with viral suppression in all three samples. In addition, fewer treatment interruptions (p = 0.0006) and more knowledge on ART (p = 0.0003) were positively associated with viral suppression in the first sample. (Table 3)

**Table 3:** Associations between socio-demographic, treatment history and viral suppression among three samples of PLWH in Guangxi.

	Sample 1 (N = 1643)			Sample 2 (N = 415)			Sample 3 (N = 168)		
**Viral load (copies/ml)**	<50	≥50	P	<50	≥50	P	<50	≥50	P
**N (%)**^**a**^	1341 (81.6)	302 (18.4)		356 (85.8)	59 (14.2)		139 (82.7)	29 (17.3)	
***Socio-demographic***									
Gender									
Male	789 (58.8)	186 (61.6)	0.38	205 (57.6)	35 (59.3)	0.80	93 (68.4)	20 (69.0)	0.95
Female	552 (41.2)	116 (38.4)		151 (42.4)	24 (40.7)		43 (31.6)	9 (31.0)	
Age (mean±SD, years)	42.0 ±12.1	43.4 ±13.0	0.08	38.3±5.5	36.9±5.9	0.08	40.9±8.4	43.2±9.7	0.21
Ethnicity									
Han	874 (65.3)	191 (63.3)	0.50	225 (67.4)	31 (55.4)	0.08	84 (60.9)	10 (35.7)	**0.01**
Others	465 (34.7)	111 (36.7)		109 (32.6)	25 (44.6)		54 (39.1)	18 (64.3)	
Education (mean±SD, years)	7.1 ±3.0	6.9±3.1	0.17	7.1 ±2.5	7.1 ±2.4	0.88	6.2 ±2.5	6.0 ±1.7	0.65
Job									
No job	326 (24.4)	97 (32.3)	**0.007**	67 (19.0)	5 (8.5)	0.08	21 (15.1)	5 (17.2)	0.55
Part-time	467 (34.9)	83 (27.7)		111 (31.4)	17 (28.8)		23 (16.6)	7 (24.1)	
Full-time	544 (40.7)	120 (40.0)		175 (49.6)	37 (62.7)		95 (68.4)	17 (58.6)	
Marital status									
Single/Separated	130 (9.9)	42 (14.4)	**0.04**	6 (1.7)	2 (3.4)	0.43	14 (10.1)	2 (6.9)	0.35
Married/Living with boyfriend or girlfriend	944 (71.7)	191 (65.4)		277 (78.0)	42 (71.2)		113 (81.3)	22 (75.9)	
Divorced/Widowed	242 (18.4)	59 (20.2)		72 (20.3)	15 (25.4)		12 (8.6)	5 (17.2)	
Household's monthly income (CNY)									
< 2000	1117 (83.8)	244 (81.3)	0.30	306 (86.0)	52 (88.1)	0.65	70 (50.4)	14 (48.3)	0.83
≥2000	216 (16.2)	56 (18.7)		50 (14.0)	7 (11.9)		69 (49.6)	15 (51.7)	
HIV transmission mode									
Sexual intercourse	947 (83.6)	206 (83.1)	0.84	239 (82.4)	46 (90.2)	0.17	113 (96.6)	27 (93.1)	0.40
Transfusion/needling sharing	186 (16.4)	42 (16.9)		51 (17.6)	5 (9.8)		4 (3.4)	2 (6.9)	
***HIV-related characteristics***									
Duration of HIV diagnosis (mean±SD, months)	50.7±27.9	39.3±27.4	**< .0001**	54.4±29.1	50.1±32.8	0.33	26.2±8.7	17.8±9.1	**< .0001**
Any co-morbidity (Yes)	202 (15.1)	49 (16.2)	0.61	61 (17.1)	11 (18.6)	0.78	14 (10.1)	3 (10.3)	0.96
***Treatment history***									
Whether on ART									
Yes	1309 (97.8)	265 (88.3)	**< .0001**	348 (97.8)	53 (89.8)	**0.0018**	139 (100.0)	28 (96.6)	**0.03**
No	30 (2.2)	35 (11.7)		8 (2.2)	6 (10.2)		0 (0)	1 (3.4)	
Duration of HIV treatment (mean±SD, months)	42.6±23.6	30.6±20.4	**< .0001**	42.3±23.2	35.3±24.2	**0.04**	25.7±7.8	17.7±8.7	**< .0001**
Treatment interruption (Yes)	21 (1.6)	13 (5.0)	**0.0006**	8 (2.4)	1 (1.9)	0.82	NA	NA	
Knowledge on HIV treatment (mean±SD)	38.5±3.7	37.6±4.0	**0.0003**	NA	NA		38.9±3.9	39.8±5.9	0.44

^a^ The number of participants for some of the variables did not add up to the total sample size because of missing data.

## Discussion

In our study, the ART coverage and rate of viral suppression (HIV RNA <50 copies/ml) were both relatively high across different time points among PLWH who received care in Guangxi. The rates are comparable with previous studies conducted in China [18, 26] and even higher than some developed countries [33]. However, the treatment outcomes were still behind the UNAIDS 90-90-90 targets [8]. The large proportion (around 50%) of lower CD4 counts (≤350 cells/mm^3^) also suggested a room for improvement in immune recovery, because the decline of CD4 count can lead to an increase of both opportunistic infections and mortality [34]. Expanding the HIV testing and improving early linkage to care could be helpful to get more PLWH on treatment before their immunological functioning become deteriorated.

The increasing trend in ART coverage and CD4 count over time might be attributed to the implementation of the updated treatment guidelines. It is worth noting that, different from existing studies, our samples were collected from community-based clinics in mostly rural settings. Compared with those studies using the data from major health care facilities, such as urban hospitals, our study showed similar clinical outcomes. This finding suggests that the HIV care can be delivered effectively in community settings, which was also supported by the research conducted in other settings [35, 36].

Our study demonstrated that female and younger PLWH had higher CD4 counts than their counterparts. This is consistent with the previous studies [37–40]. It suggests that women have a more favorable immunological pattern than men. The older populations with HIV infection tend to be in a worse stage at initial diagnosis and probably have a more aggressive clinical course compared with younger HIV-infected populations. Future studies should examine whether clinical management strategies should be different for men and older PLWH in resource-limited settings.

The higher proportion of co-morbidity with other infections (e.g., Syphilis, Hepatitis) was associated with lower CD4 counts. Previous studies showed that the co-infection of acute bacterial STI (e.g., Syphilis) resulted in increased HIV viremia and a decrease in CD4 counts [41, 42]. With the increase of CD4 counts, there was a decrease of the rate of ART initiation. This finding might reflect the evolving eligibility criteria for ART in terms of CD4 threshold in different national treatment guidelines [12, 13] over time in China (i.e., ≤350 cells/mm^3^ till May 2014 and ≤500 cells/mm^3^ till June 2016). Therefore, free ART was historically prioritized to those with lower CD4 counts, which resulted in a higher ART coverage among patients with CD4 counts ≤350 cells/mm^3^.

Our findings demonstrated that ethnic minority, unemployment, and absence of stable partners were associated with suboptimal viral suppression among different samples. Guangxi is among one of the provinces that have multiple ethnic groups. Owning to sparse population distribution and province-wide low socio-economic status, the PLWH in Guangxi generally received lower levels of education and less likely to have stable jobs. Furthermore, the participants who were not married or didn’t have a boyfriend or girlfriend might have less social support for HIV treatment compared with PLWH with stable partners. All these characteristics could affect the ART adherence [43] and therefore, compromise the viral suppression. This finding is corroborated by a different research conducted in Guangxi [20].

Our data also suggested that many challenges exist to achieve viral suppression during the course of treatment. For example, participants who had less knowledge of HIV treatment and more treatment interruptions were less likely to be virally suppressed in sample 1. ART interruptions undermine the effectiveness of ART by prompting viral load rebound [44, 45], thereby making it harder to achieve viral suppression. Treatment-related knowledge is an essential prerequisite for ART adherence. Enhancing knowledge of HIV treatment and the importance of optimal ART adherence are needed to achieve successful viral suppression among PLWH on ART.

Our study has several limitations. First, the three samples might not be totally comparable. While all three studies were conducted in similar community-based treatment settings (e.g., HIV clinics), the samples could be different because of some specific inclusion criteria in each study. Second, there might be some overlap in samples between sample 1 and sample 2 due to the similar settings and timing of the studies. However, such possibility was considered small because of the specific inclusion criteria (e.g., only parents living with seronegative children aged 6–15 years in study 2). Third, our samples consisted of PLWH who were largely engaged in HIV care or treatment. Therefore, the clinical outcomes reported in this study may not be representative of other PLWH who were not retained in care or experienced interrupted care. Fourth, all the participants were recruited in Guangxi, thus caution should be taken when generalizing our results to other regions, especially, given the fact that Guangxi has been the only region in China that has witnessed a decrease in the number of newly diagnosed HIV cases in the past six years [11]. Fifth, both CD4 counts and viral load were taken from patients’ medical records, but no information was available in terms of the laboratory procedure or accuracy. In addition, viral load data were only available from about half of the sample. Sixth, the nature of cross-sectional data cannot establish causal relationships between clinical outcomes and various demographic treatment factors.

In summary, the increasing trends in ART coverage and CD4 count across the six years reflected the positive impacts of the HIV treatment program, especially the new treatment guidelines. However, suboptimal clinical outcomes continue to be a problem, particularly among some subgroups of PLWH. Future studies should examine whether clinical management strategies should be tailored for PLWH with different sociodemographic characteristics and treatment trajectories. Expanding the HIV testing and improving early linkage to care could be helpful to get more PLWH on treatment before their immunological functioning become deteriorated, which would also result in better treatment outcomes. Helping the patients to gain more knowledge of ART, emphasizing the importance of optimal treatment adherence and reducing treatment interruptions probably could benefit the viral suppression for the patients. Future longitudinal studies may be needed to improve our understanding of potential factors that affect the clinical outcomes among PLWH in Guangxi, China and other resource-poor settings.
